# AI and mental health: evaluating supervised machine learning models trained on diagnostic classifications

**DOI:** 10.1007/s00146-024-02012-z

**Published:** 2024-08-02

**Authors:** Anna van Oosterzee

**Affiliations:** Utrecht University, Utrecht, The Netherlands

**Keywords:** Precision psychiatry, DSM, Supervised machine learning, Ground truth, Validity

## Abstract

Machine learning (ML) has emerged as a promising tool in psychiatry, revolutionising diagnostic processes and patient outcomes. In this paper, I argue that while ML studies show promising initial results, their application in mimicking clinician-based judgements presents inherent limitations (Shatte et al. in Psychol Med 49:1426–1448. 10.1017/S0033291719000151, 2019). Most models still rely on DSM (the Diagnostic and Statistical Manual of Mental Disorders) categories, known for their heterogeneity and low predictive value. DSM's descriptive nature limits the validity of psychiatric diagnoses, which leads to overdiagnosis, comorbidity, and low remission rates. The application in psychiatry highlights the limitations of supervised ML techniques. Supervised ML models inherit the validity issues of their training data set. When the model's outcome is a DSM classification, this can never be more valid or predictive than the clinician’s judgement. Therefore, I argue that these models have little added value to the patient. Moreover, the lack of known underlying causal pathways in psychiatric disorders prevents validating ML models based on such classifications. As such, I argue that high accuracy in these models is misleading when it is understood as validating the classification. In conclusion, these models will not will not offer any real benefit to patient outcomes. I propose a shift in focus, advocating for ML models to prioritise improving the predictability of prognosis, treatment selection, and prevention. Therefore, data selection and outcome variables should be geared towards this transdiagnostic goal. This way, ML can be leveraged to better support clinicians in personalised treatment strategies for mental health patients.

## Introduction

It has been widely accepted that the Diagnostic and Statistical Manual of Mental Disorders (DSM) (5-tr ed.; DSM–5-tr; American Psychiatric Association 2022), the currently used classification system for mental disorders, suffers from significant shortcomings. For years, it has been argued that the classification system does not provide a sufficient basis for treatment decisions^1^ or allow predictions about patients’ future states based on classifications alone (Tabb 2019; Hatfield et al. 2010; Graham and Stephens 2003). This significantly constrains the development of a productive mental healthcare system that can fulfil its duty of care to mental health patients (Cooper 2015).

The patients’ clinical realities are poorly reflected in the symptoms selected by the DSM classification system (Kendler 2016). Moreover, the symptomatic heterogeneity in patient groups, which is very common, makes it difficult to predict treatment outcomes for individuals within these groups. Additionally, comorbidity, the co-occurrence of multiple disorders, complicates the understanding of the problems at hand and the selection of proper treatment, causing many patients to miss out on necessary healthcare simply because they do not fit neatly into the classifications. These shortcomings cause patients to receive ill-informed interventions, remain untreated, or relapse. The more severe the symptoms and complex the cases, the more difficult it is to classify the patients correctly (Walczak et al. 2018). This causes those with the highest need for care to suffer from the system's shortcomings the most.

The inability of mental healthcare professionals to properly treat their patients and the rising number of people requesting care (over 970 million cases globally as of 2019 (GBD 2019 Mental Disorders Collaborators 2022) are shaping to be one of the biggest challenges of our time, especially in low- and middle-income countries, where treatment opportunities are scarce, and in countries where war, conflict, and poverty are aggravating the prevalence of mental disorders (Lake and Turner 2017).

The use of machine learning (ML) in psychiatry has the potential to revolutionise psychiatry and improve patient outcomes. Most of these models follow examples of successes in the medical field, such as in oncology and radiology, where impressive advancements have been made in applying ML in medical imaging (Walsh et al. 2019; Shatte et al. 2019). Even though most of these models are still in the pre-clinical developmental stage, research has shown that these algorithms are able to match clinicians' success rates in distinguishing between, for example, melanoma and non-melanoma in skin cancer or the detection of malignant nodules that indicate the presence of lung cancer (Saba 2020). Generally, these models aim to mimic expert judgements and classify patients in the same categories prescribed by the physician. A compelling example of this type of model in psychiatry is the model by Vanhollebeke et al. (2019). Here, researches have applied supervised learning models to classify depressed patients based on fMRI brain scans. They trained a classification model to distinguish between the resting-state fMRI scans from healthy participants and those from participants who have been diagnosed with major depression by psychiatrists. This approach yielded an impressively high accuracy of 79–83%. Many more studies such as these are published rapidly (Aafjes-van Doorn et al. 2021; Dwyer and Koutsouleris 2022). The studies develop models that can detect patterns that indicate the presence of disorders such as major depressive disorder (MDD), autism spectrum disorder (ASD), and schizoaffective disorders (Shatte et al. 2019). Although these results seem impressive, many of them still rely on DSM classifications to label their data and structure their outcomes. Therefore, we need to examine exactly *what* is being achieved and what these accuracy figures mean.

Many concerns have been discussed regarding implementing AI into the psychiatric field (Minerva and Giubilini 2023), ranging from changing patient–clinician relationships and how phenomena of the mind relate to biomarkers (Eyal et al. 2019; Williams et al. 2019; Köhne and van Os 2021) to how the companies that develop these models should protect data privacy and where the responsibility lies (Peralta 2023; Mosteiro et al. 2022). However, relatively little has been said about how the fundamental problems of the DSM’s classifications relate to developing new models.

In this paper, I argue that although the ML approach is very promising in medicine and oncology specifically, it is a misleading parallel for psychiatry as long as it is deployed to test for mental disorders as categorised in the DSM. Where faster and cheaper diagnostic tools can significantly benefit patients in oncology, this will only be of limited advantage in the case of patients in psychiatry. The present diagnostic instruments are relatively inexpensive, and speeding up the process might improve the process itself; however, it would not improve the patient's outcomes.^2^ It is the classification system itself that does not allow patients to receive optimal diagnosis and treatment.

Therefore, I argue that developing AI based on the DSM’s categories will not offer any real benefit to patient outcomes. Due to the descriptive nature of the DSM, there is a fundamental problem in the ground truth for psychiatric ML models, which computational techniques cannot resolve. When the models are designed to have outcomes defined in terms of diagnostic classifications, they will inherit the problems of the input data. Often, it is claimed that the problems in psychiatry could be resolved by creating more precise and validated classifications. However, I argue that supervised ML technologies do not offer the possibility of developing these classifications: supervised ML models require pre-labelling of the training data sets. Therefore, these models always depend on classifications, which means that high accuracy is misleading insofar as it is understood as *validating* the classifications, and even reinforces the classifications and thus the problems associated with them.

In the first part of this paper, I will discuss the problems with the diagnostic classifications contained in the DSM, drawing from common insights in the philosophy of psychiatry. In the second part, I will lay out in greater detail how training supervised ML works and why the ground truth is crucial for the quality of these models. In the third part, I will explain why these problems clash with the training methods of supervised models and why these models are not the correct methods to choose when developing technology to support the psychiatric diagnostic process. To conclude, I will argue that the way forward is to drop the reliance on DSM classifications. I will briefly mention and discuss a variety of approaches that circumvent this problem so that ML might still offer benefits to psychiatry and support patients suffering from mental health problems.^3^

## Shortcomings of the DSM classification system

The mental healthcare system has been constructed to rigidly adhere to the classification system proposed in the DSM. Treatment is developed especially to fit the different categories, insurance systems worldwide are built on its classifications, and virtually all research data on psychopathology is labelled according to the DSM’s distinctions (Cooper 2015). I argue that this dependency limits the efficacy of care for mental health patients, especially for complex patients who do not fit neatly into the proposed categories.

The DSM’s classifications are almost exclusively based on clinically observable behaviours instead of underlying causal mechanisms, as is common in the medical sciences; these behaviours are grouped on their high levels of covariance into symptom groups labelled ‘disorders’ (e.g. MDD, ASD, etc.) (Tsou 2016). The reliability of these classifications (how consistent the test results are) is generally acceptable: most psychopathology tests are standardised, and the inter-rater reliability is even relatively high (Buer Christensen et al. 2018) However, the lack of underlying causes^4^ reduces the current psychopathological classifications to merely descriptive “labels”^5^ with low validity [whether the results of a test represent what it is trying to represent (Cabitza et al. 2019)]; they describe collections of observable symptoms but nothing more.

The DSM classifications are notorious for symptom heterogeneity which makes individual predictions difficult. For example, two patients who are diagnosed with borderline personality disorder (BPD) can have almost entirely different symptom profiles and, therefore, require completely different treatment plans (Cavelti et al. 2021). This is not the case for more homogenous groups, i.e. groups that share many of the same features; here, predictions can be made about multiple features based on limited patient information. When we diagnose a patient as belonging to a group, we expect to know things about that group that will also be true for the newly added patient (Gorenstein 1992). For example, we know that certain types of breast cancer are highly hereditary; when someone belongs to that category, we can make predictions about the risk of earlier onset, the course of the disease, and the treatments that will have a beneficial effect (Wirapati et al. 2008). However, this is not the case for most disorders mentioned in the DSM.

Abundant overlap between the symptoms of different categories exists, undermining the DSM's efficacy. This is reflected in the high prevalence of comorbidity and ‘not otherwise specified’ (NOS) diagnoses (Fisher et al. 2015) (Amerio et al. 2015). When multiple disorders co-occur, symptoms are often more severe, quality of life and cognitive functioning are negatively impacted, and is associated with a higher suicide rate. In clinical practice, each disorder is diagnosed and treated separately. The treatment plans, therefore, become complicated, and the outcomes become more negative (Spijker et al. 2020). The DSM does acknowledge the common co-occurrence of disorders but offers no solutions, leaving those who suffer the most with the least amount of support. Transdiagnostic treatment aims to offer solutions to these complex patients. For example, pharmaceutical interventions for symptoms of one disorder can be combined with therapeutic interventions for symptoms of the other disorder. Studies show that this is more effective than traditional treatment plans that treat the comorbid disorders separately (Spijker et al. 2020).

Instead of having too many different symptoms for a single diagnosis, patients can also suffer from a very limited number of symptoms, causing them not to fulfil the requirements of any given classification. These patients are categorised as ‘not otherwise specified’ (NOS) (Fisher et al. 2015). The NOS diagnosis is most common in eating disorders where the recognised disorders anorexia nervosa and bulimia are strictly defined. The diagnosis eating disorder not otherwise specified (EDNOS) is given to eating disorders that do not fulfil these strict criteria. The diagnosis is highly prevalent, with as many as 40–90% of the eating disorder diagnoses being EDNOS. It is especially prevalent among minorities, patients with low socioeconomic status, and atypical patients such as men and elderly people.^6^ The symptoms represented in the patient group are so diverse that the classification contains little information about course, outcomes, or treatment recommendations, thereby undermining its utility as a diagnosis (Thomas et al. 2009).

These problems mean the classifications of the DSM fail to fulfil specific functions that diagnostics in medicine ought to fulfil. We expect diagnoses to guide predictions about prognosis, guide treatment selection, and inform prevention efforts. Clinical practice shows us that most DSM classifications have low predictive value, preventing patients from getting the best care. I argue that this is especially harmful to complex and atypical patients who require individualised care that does not fit the current system.

In the next section, I will elaborate on medical AI for psychiatric use. I will focus on supervised ML models, as these are the most used technique for medical AI and will explain why ground truth is crucial for their quality.

## Supervised machine learning and invalid ground truth

So far, I have argued that the DSM classification system has deep-rooted problems that disadvantage patients and limit their recovery. I claim that these inherent problems cannot be resolved with the use of AI. To explain why, I will now elaborate on how supervised ML models are trained and developed for the use of mental health diagnostics.

Supervised classification techniques [e.g. support vector machine, naïve Bayes, or decision tree (Shehab et al. 2022)] are commonly used for medical AI, including various applications in psychiatry^7^ (Shatte et al. 2019). The development of this technology has played an essential role in improving the timing and accuracy of cancer detection (although the clinical application is still limited) (Saba 2020; Bhinder et al. 2021). For example, deep neural networks are trained to classify biopsies of surgical resections. They can accurately predict whether a digitised stained slide contains cancer cells or healthy cells (AUCs > 0.99).^8^

Supervised classification techniques are not merely limited to visual data sets. They are being applied to a wide range of different types of bio-data. For example, the study by L. M. Williams et al. (2011) opts for an electroencephalogram (EEG) (the measurement of the electrical activity of the brain) as input data as a possible data source to build models to classify healthy and major depressive disorder (MDD) patients. The research performed by Pham et al. (2013), explores how to analyse photoplethysmography (measuring blood volume at the surface of the skin) using nonlinear dynamical analysis, which could function as a non-invasive way to diagnose depression. These all follow the same principle: the outcome variables share the same feature of being straightforward classifications of DSM categories. The question we ask the model is, “Is this disorder X? Yes? or No?”.

Although the specificities of neural networks are complex^9^ and the data used is diverse, the process of training these models is relatively straightforward. The process follows the following steps: first, the data is collected and labelled. For example, fMRI scans of brain tumour patients are collected and labelled with the corresponding diagnoses, e.g. ‘glioma I–IV’ or ‘healthy scan’. Then, the data is pre-processed to reduce noise and decrease the risk of overfitting (Bhinder et al. 2021). Next, the data set is split into the training and testing sets. The model is selected, and the training set is used to teach the model to classify between the pre-set classifications based on patterns in the given data. It learns which image belongs to which label. In other words, the AI is trained to look at data and classify it into predefined output classes, e.g. ‘health’ and ‘disease’. As a last step, the model is evaluated by showing it an unlabelled version of the testing set to test how well the algorithm classifies these new images. This evaluation is expressed using a confusion matrix, which includes true positives, false positives, true negatives, and false negatives. It can also be used to calculate accuracy, recall, specificity, and precision (Hicks et al. 2022).

The pre-labelling of the data set is done by human professionals^10^ and shows the model the correct label and what is, therefore, to be reproduced when encountering new data. The quality of these data sets determines the quality of the algorithm's performance. When there is e.g. bias or noise in the data, the risk is that the model will simply reproduce this in its output. In medical AI, these labels are produced by physicians who receive unlabelled data sets (e.g. fMRI scans), sometimes, but not always, accompanied by further patient data and additional tests. They are asked to cast their expert judgement and to diagnose the patients based on the given data. These diagnostic judgements are the labels that make up the “ground truth” data set on which the model will be trained. This data set is referred to as the ground truth, as it represents the real-world ‘truth’ of the data to which the AI otherwise has no access (e.g. a picture of a dog with the label ‘dog’ and a picture of a cat with the label ‘cat’). It is essential to realise that a range of factors can influence medical judgement: human mistakes, biases, missing data, disagreements, etc. (Cabitza et al. 2019). Therefore, the ground truth set is bound to include some level of uncertainty. This can be remedied by, for example, having multiple physicians cast their judgements on the same data and create the ground truth set based on their average judgement. Or to include a three-month follow-up to validate whether the diagnosis was correct so the ground truth set can be constructed by using only the validated diagnoses (Lebovitz et al. 2021).

Recent research has focused on ensuring the highest possible quality of these ground truth data sets. This is necessary because this data set determines the quality of the model's outcome. Supervised ML models are, in principle, “expert mimicry” systems: they are optimised to reproduce the judgements of the experts they are trained on. If the experts' judgement is unreliable, the model will be unreliable. In the next part of this paper, I will argue that this dependency on the quality of the labelling set causes problems when developing models for psychiatry.

## The shortcomings of expert mimicry systems

Now that I have elaborated on the DSM classifications and have a general understanding of supervised ML models, we can return to the question: *What* is achieved when ML algorithms classify patients into disorder categories based on biomarker data?

I argue that although it might seem that ML models improve the outcome of the diagnostic process, they are not able to provide an output variable that is a more valid or predictive classification than the psychiatrist’s classifications. The models that are being developed for psychiatry are ‘expert mimicry systems’: they are trained on a ground truth labelled by experts, and the outcome of the model mimics what the expert would have said when they would have seen the data. Given that the experts use the DSM’s diagnostics classifications to label the patients, the model is bound to inherit the validity and prediction problems related to these labels. Whether a patient receives the diagnosis through a psychiatric consult or the AI system, the outcome will be the same. The diagnosis given by the AI will have the same low predictive validity that the experts’ diagnosis would have had. The patient will receive a descriptive diagnosis that only describes their symptoms, which were simply observable in the first place, and nothing else. The addition of the ML model will not have altered the outcome in any meaningful way.^11^ Generally, these types of problems in AI are known as “garbage in, garbage out” problems. When the input data is of poor quality, there will be problems in the outcome variables (Kilkenny and Robinson 2018). However, where cleaning the data is often the suggested solution (getting rid of noise, biases etc.) in the current situation, this will not work. It is not possible to ‘clean’ low validity.

These problems are not unknown (Stephan et al. 2017), yet many studies still strive to improve reliability, validity and predictability while using a training set labelled on a DSM-based ground truth. Therefore, the warning deserves rehearsing. For example, in Veld Mohammadi et al. (2015), EEG data is implemented to classify healthy and major depressive disorder (MDD) patients. Interestingly enough, they acknowledge the disorder’s heterogeneous nature and that diagnosing depression currently presents a clinical challenge. Nevertheless, they still use the clinically labelled variable MD, which will inevitably give their newly found pattern little predictive value as a biomarker.

Now, you might wonder if the missing pathological causal pathways are the problem. Could these ML models not improve the validity of psychiatric classifications by identifying the patterns in the data that are related to the underlying pathways? It is indeed true that these pattern recognisers are exceptionally good at recognising patterns. Additionally, these models use a different data source than traditional diagnostics. Psychiatrists use questionnaires and behavioural observations, while the models often use bio-data. I argue that the problem is that we would never know whether we had found a validating pattern when using these models.

Let me elaborate: The optimisation process of ML models aims to achieve 100% accuracy based on the given training set. This means that when a clinician labels a specific patient as ‘depressed’ based on the outcome of their diagnostic tools, and the algorithm labels the same patient depressed based on its model, this is considered a true positive. Hence, we derive accuracy measures that tell us how closely the output resembles the training set (Orrù et al. 2012). To mimic this expert’s judgement, the model searches for patterns in the data. In doing so, the algorithm is expected to latch onto patterns in the data that are similar for patients in the same labelled category, but different across the categories. Given that the difference between the categories is the presence of the disorder, the pattern in the (bio)data is expected to be related to a biological aspect of the disorder, possibly an underlying mechanism. Psychiatrists are aware that this underlying pattern will not follow the exact judgements of the clinicians who labelled the data. As explained before, the DSM classifications are, in reality, not clean-cut, even if they appear that way.^12^ Due to heterogeneity, there is sizeable intra-group variability, and due to comorbidity, there is also considerable overlap between the to-be-distinguished categories. This situation would mean that when the underlying pattern exists, it would not be found in all patients who are labelled with the disorder. Some of the ‘depression’ labels are, in fact, false positives on the clinician’s side, and some of the ‘healthy’ labels are, in fact, false negatives. Unfortunately, therefore, the model that has found the ‘correct’ pattern will receive a low accuracy measure (remember a true positive is when both the ML model and the psychiatrist labelled the data as depressed). How well the model performs is judged on the original labelling set.

To establish whether the AI recognised a “pathological causal pattern” in the data, we would need a second ground truth set, an “underlying truth “, which was labelled based on this underlying mechanism. However, as science has not discovered these mechanisms, this knowledge is currently inaccessible to us.^13^ Therefore, even if we could observe the pattern found by the model (with explainable AI,^14^ for example), we could not determine whether this is genuinely related to the pathology. This means that, for now, we can only derive accuracy measures that tell us how closely the output resembles the psychiatrist’s judgement and not how closely it resembles an underlying mechanism. Therefore, I argue that high-accuracy measures are misleading insofar as they are understood to validate the outcome classifications because high accuracy means that the heterogeneity that causes low validity is mimicked.^15^ In the last part of this paper, I will lay out how supervised ML models could be used more fruitfully in psychiatry when the outcomes are focused specifically on improving the predictability of prognosis, treatment selection and prevention.

## Part 4 Predictive labels for prognosis, treatment, and prevention

So far, I have argued that the current classification system provided by the DSM suffers from significant shortcomings, which constrain patients’ recovery chances. The system especially disadvantages minorities and those with the most complex symptom profiles. When AI is developed that uses these classifications in the labelling of their data,^16^ it will inherit the existing problems and further lock in an already rigid healthcare system, preventing psychiatry from moving beyond its current shortcomings. However, this does not mean that I am pessimistic about developing AI systems for mental healthcare. When models are developed that focus on improving the predictability of prognosis, treatment selection and prevention instead of on predicting DSM classifications, it could greatly benefit patient outcomes. In this last section, I will highlight a few examples of more predictive labels and alternative approaches to developing diagnostic tools and discuss their advantages and disadvantages.

The clinical practice uses many classifications that have greater predictive power might than official diagnostic categories, which could be used to train ML models. An example of these are the classifications used for suicidal-related behaviour, i.e. ‘suicidal ideation’ or ‘suicidal attempt’, which describe concrete behaviours or cognitions that can be observed or measured [by using, e.g. the Columbia Suicide Severity Rating Scale (Posner et al. 2011)]. The suicidal detection model that has been developed by Ophir et al. (2020) uses these classifications to label their data to improve prevention strategies for suicidal patients. Ophir et al. developed a deep neural network to predict suicidal tendencies based on social network content. Because these labels describe behaviours that can be measured or observed instead of latent variable classifications that describe themselves, the ground truth of these models could possibly be validated. In this case: a second training set could be established based on predicted suicide attempts that actually took place. Because of this validation, these models could achieve more reliable predictions for suicidal tendencies than current psychiatric practices, which are currently only slightly better than chance (Franklin et al. 2017). The development of these models, however, does raise ethical concerns that ought to be taken seriously. The collection of such sensitive data requires great care, and privacy should be in the foreground. Additionally, there is a high risk of bias and false positives and negatives. As ML is prone to bias, it should be carefully considered how to make sure that populations are not under or overrepresented in the data sets and, therefore, are flagged too often or not often enough when their mental health declines, which could aggravate social injustice and limit access to healthcare.

Another example of predictive labels is used by the start-up “Predictix” (*PREDICTIX*®* By Taliaz*, n.d.), which focuses on improving treatment selection for patients suffering from mood disorders. The team developed a model that uses genetic information to predict the best choice of antidepressant-type medication (Taliaz et al. 2021). Currently, when depression is diagnosed, there is no good way to predict which of the available antidepressant treatments will be most efficient for the patient. Most patients will enter a long and tedious process of trial and error to find which medicine levels will alleviate their symptoms. Given that the diagnosis is not helpful in this process, a biomarker that does not describe a pathological process but is only concerned with the functioning of the medication can significantly benefit the patient without having to be concerned with the predictive validity of the diagnosis. However, here, too, the downsides must be considered. The medicalisation of mental disorders is met with great resistance. Most antidepressants barely perform better than a placebo, and often psychological and environmental factors play a large role in mental suffering, which is not resolved by the medication (Hengartner 2022). When antidepressants become easily available, there is a risk that the healthcare system shifts further away from doing the hard work to improve someone's mental health to easy and quick fixes, made even easier with the help of AI.

Alternatively, there is the possibility to look beyond the current system in search of predictive labels.^17^ Research has shown that many of the DSM’s categories are, in fact, dimensional, and the thresholds (i.e. symptom is not present/symptom is present) are arbitrary ones (Maj 2018; Hengartner and Lehmann 2017). This causes many patients to fall right below the threshold, even though they do suffer significantly from their symptoms. The Hierarchical Taxonomy of Psychopathology (HiTOP) (Kotov et al. 2017) is a consortium that aims to develop a new nosology of psychopathology to address this problem. Similarly to the DSM, it is atheoretical and focuses on symptom covariance. However, instead of viewing disorders as discrete conditions, HiTOP views them as continuations of normal behaviour. Therefore, HiTOP’s constructs are dimensional. Additionally, HiTOP focuses the data collection that is used to construct their classifications on a more diverse population, including non-Western patients and young children (Kotov et al. 2022), which improves the generalisability of models built upon these classifications. Using HiTOP’s classifications to train ML data instead of the DSM classifications could circumnavigate certain problems present in traditional diagnostics. However, as HiTOP’s classifications are constructs, the models trained on them will run into the same ground truth problem as those trained on the DSM’s classifications.

Another alternative is the Research Domain Criteria (RDoC) project, which, similarly to HiTOP, adopts a dimensional approach. However, it differs from HiTOP and the DSM in that it does not follow a symptom-based definition of disorders; it aims to create a nosology based on pathophysiological processes and observed behaviour (Cuthbert and Insel 2013). This could possibly resolve the ground truth issue, as a pathophysiological process could be used as a means of validation. When a model is trained on RDoC labels, the predicted disorder could be validated by the presence of the underlying process. However, the RDoC nosology is currently developed for research purposes. The physiology of mental disorders is still poorly understood, and it could take decades until this knowledge is developed far enough to be used in clinical practice. Nevertheless, the framework has proven to be a great inspiration for computational psychiatry research where currently, high-dimensional data sets are being deployed to combine behavioural, symptomatic, and physiological features (Cuthbert 2020).

These examples demonstrate that there is much to gain when research focuses specifically on improving the predictability of prognosis, treatment selection and prevention. Therefore, data selection and outcome variables should be geared towards this transdiagnostic goal. For all these applications, it is important to consider the technical possibilities and the societal implications. Data collection runs the inherent risk of biases. With continuous data collection, it is crucial to consider privacy and agency, especially regarding sensitive data such as health data. I argue that this requires careful consideration moving forward. On the other hand, new tools may also serve important social values, like health equity. Healthcare systems around the world struggle with the enormous challenge of providing services and support to those most in need. The DSM has proven to be a poor instrument to address these difficult distribution questions. ML instruments, particularly when made widely available in online form and trained with the right labels and categories, could make an important contribution to getting health services to those most in need.

## Conclusion

Precision psychiatry is a growing field, and supervised ML is one popular approach to developing tools to aid in the diagnostic process. In this paper, I have argued against using DSM categories for these models. Due to the heterogeneous nature and the abundant comorbidity of disorders, supervised ML models trained with these labels will have low validity and little predictive value. This problem cannot be solved due to the inaccessible ground truth.

I have argued that it is impossible to develop models that do not inherit these problems. The reason for this is twofold. First, the model is optimised on a DSM-based ground truth provided by clinicians; it is impossible to achieve a higher predictive validity than the original clinicians could with DSM classifications alone. A supervised model cannot be more valid than its training data; it can only aim to mimic the expert exactly. Secondly, the lack of underlying mechanisms results in an inaccessible “underlying truth”; therefore, it is impossible to verify whether a model has found a pattern related to a pathological causal mechanism in the heterogeneous patient group. This means that high-accuracy measures are misleading when they are understood to validate the models’ outcomes.

Therefore, the model will inherit the problems caused by the DSM system, which limits patients' recovery chances and especially disadvantages those worse off. When ML models are trained on more predictive data sets, such as those focusing on treatment outcomes and less on diagnostic categories, they can provide clinicians with tools to support their patients. However, careful consideration is needed to avoid rehashing past mistakes when selecting these data sets and the chosen labels.

## Data Availability

No data was used in this study.

## Footnotes

1 For the most common psychiatric treatments (i.e. cognitive behavioural therapy), it is irrelevant which disorder the patient is categorised into; recovery rates are very similar across the different DSM classifications (Cuthbert and Insel 2013). The therapeutic alliance, for instance, predicts outcomes much more effectively than anything else.

2 Adding an expert mimicry system as a second opinion might offer some interesting benefits. It enhances the reliability of the outcomes and could improve the trust between the patient and the therapist; on the other hand, it could also show biases or prejudices and help to circumvent those, for example, in the case of systematic underdiagnosis.

3 I have written this paper with the mental health patients as the primary stakeholders, who are meant to benefit from the diagnostic process and therapy choices based on them. There will be other stakeholders, such as tech companies, insurance companies, policymakers, and epidemiologists, who might have diverging interests, but I shall process on the assumption those are secondary to patient health.

4 During the decades after the introduction of the DSM-III, various neural and genetic mechanisms have been discovered that play a role in the symptoms of these mental disorders. However, few of these discoveries can offer full explanations of the disorder and all its symptoms. Instead, it is becoming increasingly clear that for most mental disorders, the pathophysiological underlying mechanisms are heterogeneous in nature, and it is unlikely that a common cause will ever be found (Murphy 2012).

5 “Label” is often colloquially used to describe a diagnostic classification “to give a child the label ADHD”. However, due to the use in the machine learning literature, “label” in this paper will refer to the labelling of data sets, which could be diagnostic classifications, but also medical or merely descriptive (i.e. the label ‘tree’ or ‘dog’).

6 Another example of atypical patients that are being disadvantaged is the systematic underdiagnosing of women when it comes to ASD (autism spectrum disorder (Zener 2019).

7 There are other techniques that are considered ‘AI’ that are being explored for use in the medical and psychiatric setting, such as unsupervised learning techniques or causal modelling. Although these too show promising results, they are currently outside of the scope of this paper and will not be discussed.

8 AUC is a commonly used measure to judge the performance of a model. AUC stands for area under the receiver operating characteristics curve, which indicates the trade-off between sensitivity and specificity. An AUC of > 0.80 is considered good, but it is still up for debate whether this is also a clinically acceptable threshold (Bhinder et al. 2021).

9 Recently, convolutional neural networks (CNN) have become the most popular deep learning architectures used for image classification in medicine, in which the workflow is a little different from more old-fashioned image classification MLs. To train these networks on medical images, transfer learning is an often-used approach. It uses a large collection of natural objects to train the initial layers of a model (the models learn to identify general things, such as shapes and colours). Secondly, it uses specific medical data to fine-tune the last layers (Bhinder et al. 2021).

10 The labour that goes into manually labelling these data sets has been a recent topic of debate, especially in light of Amazon's ‘Mechanical Turk’, which vastly underpays workers who label visual data sets.

11 Expert mimicry systems could be beneficial in situations where no trained professionals, or only poorly trained professionals, are available for the diagnostic process. In these cases of absence, the model could offer support by replacing this process.

12 For an example, see the study by Maciejewski et al. (2016), which shows that the classifications of prolonged grief disorder, complicated grief, and bereavement disorder (which are distinct categories according to the DSM) almost overlap entirely.

13 There is a lively debate that argues that underlying mechanisms have not been found because they are non-reducible; see Borsboom et al. (2019). This debate, however, is outside the scope of this paper. Moreover, the outcome would not change the argument at hand.

14 Often it has been argued that ML models are ‘black box’ models, because it is difficult to uncover how these models make the decisions they do. Explainable AI tools are being developed to give us more insight into these opaque ML models (Farahani et al. 2022)(Zednik and Boelsen 2022).

15 Not only might these models be misleading to the researchers developing them, but also to the clinicians and the patients. The impressive process of fMRI or EEG scanning and the quantitative outcome of an AI model could lead patients to believe that the diagnosis is more valid than it is. A patient might be led to think: “Now that they have seen it in my brain, it must be true.” By creating a bio-technical diagnostic process, we risk creating the illusion of a valid biomarker that can be reliably applied in the clinical setting (Lakhan et al. 2010). However, when the outcome of the model is, in fact, the same classification that the psychiatrist is using, nothing will change for the patients’ outcome.

16 It is also important to consider that the labels that are chosen can have a large societal impact. Many mental health-related concepts carry a stigma, and when they are selected to train models, this stigma should be taken into account, especially when there is a high risk of false positives. The classification ‘schizophrenia’, for example, carries a public stigma; patients are often viewed as ‘dangerous’ or ‘psycho’, and being classified as schizophrenic can influence someone's self-image, social life and career opportunities. Additionally, many disorders carry self-stigma, such as ADHD, ASD and personality disorders. Stigma limits access to healthcare, increases self-blame and has negative effects on recovery rate (Walsh et al. 2020).

17 Additionally, breaking open the diagnostic space could also allow alternative views to the Kraepelin view on mental health, such as 4e cognition approaches that focus on dynamics, environment and empathic understanding (de Haan 2020). An exciting example of this is the work by Northoff and Hirjak (2023) that combines approaches from cognitive neuroscience and phenomenology to build a system that does right by the patient’s lived experience.
